# Appendiceal bleeding in an elderly male: a case report and a review of the literature

**DOI:** 10.1186/s40792-021-01234-3

**Published:** 2021-06-23

**Authors:** Yuto Maeda, Seiya Saito, Mayuko Ohuchi, Yuka Tamaoki, Jiro Nasu, Hideo Baba

**Affiliations:** 1 Department of Gastroenterological Surgery, Graduate School of Medical Sciences, Kumamoto University, 1-1-1 Honjo, Chuo-ku, Kumamoto, 860-8556 Japan; 2 Department of Surgery, Kumamoto Chuo Hospital, 1-5-1 Tainoshima, Minami-ku, Kumamoto, 862-0965 Japan

**Keywords:** Lower gastrointestinal bleeding, Appendix bleeding, Appendectomy

## Abstract

**Background:**

The prevalence of acute lower gastrointestinal bleeding has been increased including colonic diverticulitis and angioplasty. However, appendiceal bleeding is extremely rare.

**Case presentation:**

We present a case of lower gastrointestinal bleeding from the appendix in an elderly male who presented with melena. Appendiceal bleeding was diagnosed using lower gastrointestinal endoscopy, and laparoscopic appendectomy was performed. The patient did not have melena postoperatively, and was discharged 6 days after the surgery.

**Conclusion:**

It is important to distinguish appendiceal bleeding from lower gastrointestinal bleeding and to treat it as soon as possible with less invasiveness.

## Background

Acute lower gastrointestinal bleeding has been increased, including colonic diverticulitis and angioplasty [1–3]. However, the source of appendix bleeding is very rare. We have experienced a case in which the source of lower gastrointestinal bleeding was the appendix in an elderly male. Here, we present a case studies to treat appendiceal bleeding, and we report it with some review of the literature.

## Case presentation

**Fig. 1:**
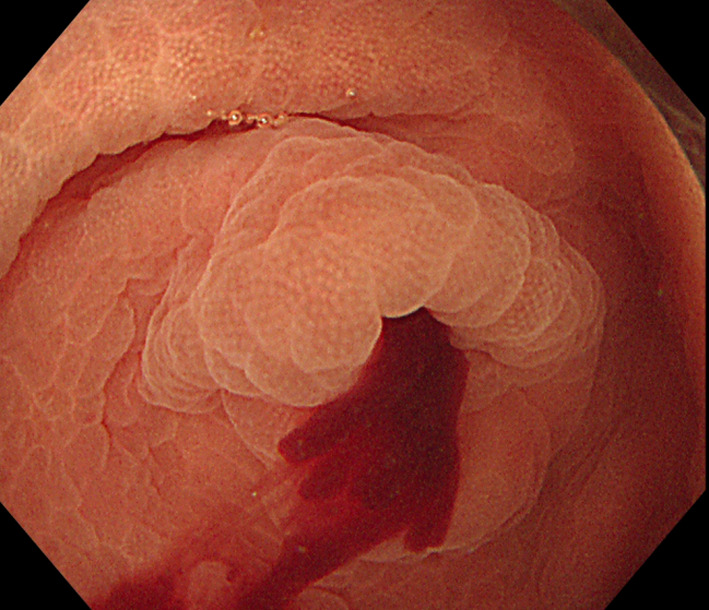
Lower gastrointestinal endoscopic findings. Bleeding is observed from the appendiceal orifice

**Fig. 2:**
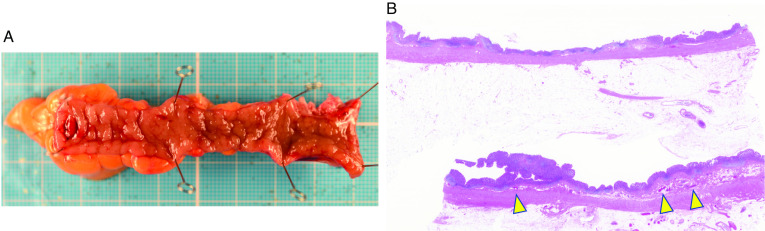
Excised specimen, histopathological findings. **a** Appendix is 70 × 5 mm in size. No abnormalities were observed on the mucosal surface. **b** Pathological findings showed a couple of bleeding points into the submucosa (arrow head), but no rupture of the arterial wall or abnormalities on the mucosal surface were observed

A 90-year-old man presented to our hospital with melena that lasted for about 2 days. There were no signs of hematochezia. No gastrointestinal symptoms such as abdominal pain were observed. He had no apparent family history of colorectal cancer. He had been diagnosed with hypertension, benign prostatic hyperplasia, and atrial fibrillation, and was taking bayaspirin for atrial fibrillation. The patient had no fever or abdominal pain, and his conjunctivae were not pale. Nevertheless, a digital rectal examination showed blood clot at the time of consultation at our hospital. On admission, laboratory evaluation revealed anemia with hemoglobin of 11.9 g/dl and a hematocrit of 34.9%. Lower gastrointestinal endoscopy was performed, but the cause of the bleeding could not be identified. However, 1 day following admission, he passed dark stools again. One day later, his hemoglobin level dropped from 11.9 g/dl to 10.4 g/dl; therefore, an emergency lower gastrointestinal endoscopy was performed. This showed that bright red blood was oozing out of the appendix. Immediately after washing out the blood, appendiceal bleeding was evident (Fig. 1). Next, laparoscopic appendectomy was performed. During the surgery, the appendix was 70 × 5 mm in size and there were no inflammatory changes or adhesions seen in the appendix. No abnormalities were observed on the mucosal surface; however, bleeding was observed from the appendiceal wall. On histopathology, we observed a couple of bleeding point into the submucosa, but diverticula or neoplastic lesions that may have caused the appendical bleeding were not found (Fig. 2). One day following surgery, no melena was observed and the patient was discharged from the hospital 6 days after the surgery.

## Discussion

Lower gastrointestinal bleeding is a common cause of hospitalization. Patients with lower gastrointestinal bleeding usually require a blood transfusion and interventions, such as gastrointestinal endoscopy and surgical treatment. Common causes of lower gastrointestinal bleeding include diverticulum bleeding, ischemic colitis, angioectasia, and post-polypectomy bleeding. Other less common causes include rectal ulcerative colitis, infectious colitis, inflammatory bowel disease, colorectal polyps / neoplasms, radiation proctitis, and hemorrhoids. However, the bleeding from the appendix is a very rare cause of lower gastrointestinal bleeding [4, 5]. This is despite the fact that the same factors that are responsible for lower gastrointestinal bleeding (i.e., inflammation, angiodysplasia, diverticulum, granulomatous appendicitis, tumor and damage of the appendix mucosa) can also cause appendiceal bleeding [6–8]; however, the cause of appendiceal bleeding may not be identified. A search on PubMed/MEDLINE database for literature published between January 1977 and August 2020 regarding “appendix bleeding” or “appendix hemorrhage” identified 30 articles [9–36] (Table 1). The average age of cases of appendiceal bleeding was 46.6 years (14–90 years). Our case was diagnosed at the oldest age among the cases, and the treatment was effective.

**Table 1:** Case of appendiceal bleeding

Case	Age	Sex	Clinical findings	Treatment	References	Case	Age	Sex	Clinical findings	Treatment	References
1	46	Male	Appendicitis	Appendectomy	[10]	16	48	Male	Diverticular hemorrhage	Appendectomy	[24]
2	33	Male	Diverticulitis	Appendectomy	[11]	17	14	Male	Appendix abscess	Ileocaecal resection	[25]
3	72	Male	Angiodysplasia	Appendectomy	[12]	18	24	Male	Granulomatous appendicitis	Appendectomy	[26]
4	22	Male	Granulomatous appendicitis	Appendectomy	[13]	19	49	Male	Acute suppurative appendicitis	Appendectomy	[27]
5	68	Male	Appendiceal dieulafoy lesion	Appendectomy	[14]	20	63	Male	Diverticulitis	Hemicolectomy	[28]
6	44	Male	Diverticulitis	Hemicolectomy	[9]	21	18	Female	Intussusception	Appendectomy	[29]
7	51	Male	Dieulafoy lesion	Appendectomy	[15]	22	33	Female	Cause unknown	Ileocaecal resection	[30]
8	71	Male	Appendix ulcer	Barium enema	[16]	23	36	Male	Intussusception	Appendectomy	[31]
9	41	Male	Atypical florid vascular proliferations	Appendectomy	[17]	24	38	Male	Aortoenteric fistula	Appendectomy	[32]
10	59	Female	Aortoenteric fistula	Hemicolectomy	[18]	25	49	Female	Appendix cancer	Ileocaecal resection	[33]
11	25	Male	Focal erosion of appendix mucosa	Appendectomy	[19]	26	53	Female	Appendicitis	Appendectomy	[6]
12	42	Male	Appendiceal mucosal erosion	Appendectomy	[20]	27	44	Male	Cause unknown	Appendectomy	[34]
13	56	Male	Gastrointestinal stromal tumor	Appendectomy	[21]	28	33	Male	Cause unknown	Appendectomy	[35]
14	76	Female	Angiodysplasia	Appendectomy	[22]	29	70	Male	Cause unknown	Colonoscopy	[36]
15	32	Female	Ulcerated appendiceal stump	Appendectomy	[23]	our case	90	Male	Cause unknown	Appendectomy	

The pathological findings in our case showed a couple of bleeding points into the submucosa, but no abnormality was observed on the mucosal surface. Furthermore, multiple diverticula or arterial wall rupture, which are thought to be the cause of appendiceal bleeding, were not found. Hence, pathological findings caused unknown of appendiceal bleeding.

In previous some reports, not only endoscopy but also CT examinations are performed for diagnosis [9–36]. However, most of the definitive diagnosis were endoscopic findings of bleeding from the appendiceal orifice. In this case as well, a definitive diagnosis could be obtained by endoscopic findings, as in previous reports. Therefore, the operation was performed as soon as possible without adding CT examination**.**

Although emergency surgery such as laparoscopic appendectomy or laparoscopic ileocecal resection is usually performed as a treatment, a strategy for appendiceal bleeding has not been established. Other treatment for appendical bleeding has been reported including arterial embolization, therapeutic barium enema, and clipping [16, 22, 36]. On the other hand, the risk of severe appendicitis, perforation and rebleeding from these treatments has been reported [37]. Therefore, surgery as a definitive treatment were performed in many cases, consecutively. In this case, although he was very eldery, we decided that laparoscopic surgery was more appropriate because of its tolerability and curability of treatment. Treatments for appendiceal bleeding should be considered as early as possible, with less invasiveness, but further research regarding its safety and complication associated with the procedure should be conducted in the future.

## Conclusions

We present a case of appendiceal bleeding in an elderly male patient. It is important to distinguish appendiceal bleeding from lower gastrointestinal bleeding and to treat it as soon as possible with less invasiveness.

## Data Availability

Not applicable.
